# Resting-State Functional Connectivity Changes in Older Adults with Sleep Disturbance and the Role of Amyloid Burden

**DOI:** 10.21203/rs.3.rs-2547880/v1

**Published:** 2023-02-09

**Authors:** Hyun Kim, Xi Zhu, Yiming Zhao, Sophie Bell, Philip Gehrman, Daniel Cohen, Davangere Devanand, Terry Goldberg, Seonjoo Lee

**Affiliations:** Columbia University; Columbia University Medical Center; Columbia University

**Keywords:** Alzheimer’s disease, resting state functional connectivity, sleep disturbance

## Abstract

Sleep and related disorders could lead to changes in various brain networks, but little is known about the role of amyloid β (Aβ) burden-a key Alzheimer’s disease (AD) biomarker-in the relationship between sleep disturbance and altered resting state functional connectivity (rsFC) in older adults. This cross-sectional study examined the association between sleep disturbance, Aβ burden, and rsFC using a large-scale dataset from the Alzheimer’s Disease Neuroimaging Initiative (ADNI). Sample included 489 individuals (53.6% cognitively normal, 32.5% mild cognitive impairment, and 13.9% AD) who had completed sleep measures (Neuropsychiatric Inventory), PET Aβ data, and resting-state fMRI scans at baseline. Within and between rsFC of the Salience (SN), the Default Mode (DMN) and the Frontal Parietal network (FPN) were compared between participants with sleep disturbance versus without sleep disturbance. The interaction between Aβ positivity and sleep disturbance was evaluated using linear regressions, controlling for age, diagnosis status, gender, sedatives and hypnotics use, and hypertension. Although no significant main effect of sleep disturbance was found on rsFC, a significant interaction term emerged between sleep disturbance and Aβ burden on rsFC of SN (β=0.11, P=0.006). Specifically, sleep disturbance was associated with SN hyperconnectivity, only with the presence of Aβ burden. Sleep disturbance may lead to altered connectivity in the SN when Aβ is accumulated in the brain. Individuals with AD pathology may be at increased risk for sleep-related aberrant rsFC; therefore, identifying and treating sleep problems in these individuals may help prevent further disease progression.

## Introduction

Alzheimer’s disease (AD), a devastating neurodegenerative disease with a high mortality rate, is currently estimated to affect 6.07 million older adults in the United States, a number that is expected to increase 18% by 2025^1^. Elucidating underlying neural mechanisms of AD through brain-based biomarkers and their interactions may advance clinical trial design for AD treatments by informing sample selection and optimizing timeframes for early intervention.

Resting state functional connectivity (rsFC) (measured via resting-state functional magnetic resonance imaging (rs-fMRI) has been utilized to identify aberrant functional architecture of brain networks that are associated with AD pathology^2^. Network analyses allow identification of aberrant coactivation of brain regions within a large-scale network, which is believed to reflect the underlying neural mechanisms of various cognitive and affective processes^2–4^. Several networks are particularly relevant to processes affected in AD. The default mode network (DMN), including lateral parietal, precuneus, and medial prefrontal regions, has been studied extensively in AD literature^5, 6^. The DMN is implicated in autobiographical memory, episodic memory, and social cognition and has anatomical overlap with regions of early amyloid-beta Aβ) accumulation, suggesting a strong association with AD pathology^7–9^. Recently, other key networks such as the frontal-parietal network (FPN; also referred to as central executive network) and the salience network (SN) also gained attention and have been shown to be altered in AD^10^. The FPN consists of the lateral prefrontal cortex and posterior parietal cortex and is responsible for various aspects of cognitive control, such as working memory, attention, and decision making^11, 12^. The SN involves the anterior cingulate cortex, anterior insula, rostral prefrontal cortex, and supramarginal gyrus, which contributes to processing emotional information and modulating activities of the DMN and FPN^13–15^. While hypoactivation in the DMN and FPN was indicative of worse progression of AD^10, 12, 16^, other studies have shown AD-related *hyperactivation* in SN, depending on the disease stages^9, 12^. Recent studies highlighted that there are periods of hyperactivity in DMN and SN in the very early stages of prodromal AD followed by hypoactive states in the later stages of AD pathology, indicating a non-linear association between rsFC and progression to AD^17–19^.

Poor sleep disturbance and related sleep disorders are well-established risk factors for AD pathology and similarly are associated with dysfunctional neural networks^20, 21^. Insomnia, for instance, is associated with decreased rsFC within the DMN but increased connectivity in the SN, particularly in the insula^4, 21–23^. A recent study has also shown that improving sleep through light therapy decreased rsFC in the SN, indicating that SN hyperactivity is an aberrant neural activity that could stem from poor sleep and that treating sleep disturbance could “normalize” SN connectivity^24^. Together, these findings indicate that sleep disturbance is associated with AD-like changes in brain activity and provide further evidence to support that improving sleep could reduce these aberrant changes in the brain.

Nonetheless, only pair-wise associations among sleep, AD biomarkers, and rsFC have been examined rather than a comprehensive picture of their interactions. While sleep-induced changes in rsFC may be impacted by AD pathology, no previous study has examined how sleep disturbance in late-life interacts with AD pathology, measured by amyloid-beta (Aβ). Aβ burden is linked to altered rsFC in older adults^25,26^, and studies have shown that functional connectivity in the DMN is disturbed in preclinical adults with Aβ positivity even before the onset of clinical symptoms^25^. Additionally, sleep burden is bidirectionally associated with increased Aβ accumulation^27^ and may moderate the relationship between Aβ and brain functions^27, 28^. Examining the interplay between sleep disturbance and Aβ would be important given their similar aversive impacts on brain aging and their potential synergistic effect in dysregulating brain network functioning. It is also crucial to examine this topic with epidemiologic data that represents older adult groups across various cognitive stages (preclinical to clinical AD).

Therefore, the current study aimed to examine the associations among sleep disturbance, Aβ accumulation based on positron emission tomography (PET) imaging, and rsFC in older adults in various stages of AD (cognitively normal, MCI, and AD) using a large-scale dataset from Alzheimer’s Disease Neuroimaging Initiative (ADNI). We first tested the association between sleep disturbance and rsFC, then evaluated the association between Aβ burden and rsFC, lastly, we assessed the interaction between sleep disturbance and Aβ on rsFC. Given previous findings on this similar topic, we hypothesized that 1) sleep disturbance will be strongly associated with altered rsFC and 2) the presence of Aβ burden in sleep disturbance will exacerbate these changes.

## Materials And Methods

### Participants:

**Figure 1:**
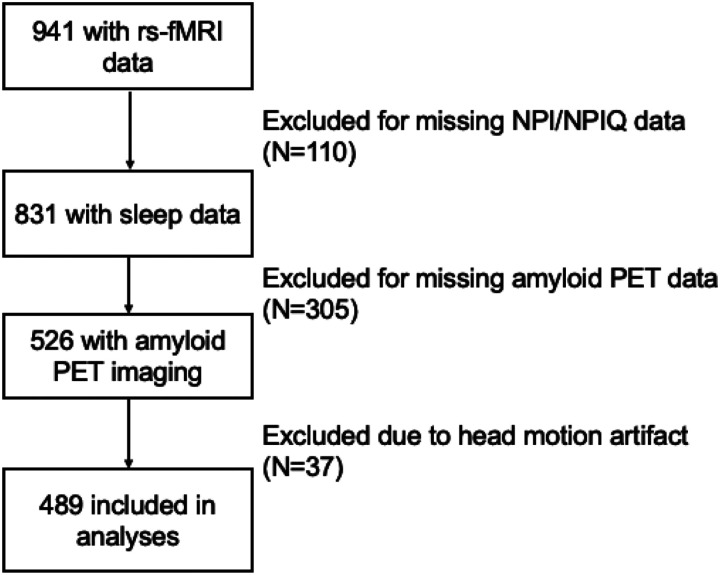
Flow chart of the study sample rs-fMRI=resting-state functional magnetic resonance imaging; NPI/NPIQ=Neuropsychiatric Inventory/Neuropsychiatric Inventory Questionnaire; PET=positron emission tomography

Rs-fMRI data were acquired from 489 participants, 262 NC (53.6%), 159 MCI (32.5%), and 68 AD (13.9%) from the Alzheimer’s Disease Neuroimaging Initiative (ADNI) (www.adni.loni.usc.edu) (Fig. 1). ADNI was launched in 2003 as a public-private partnership and examines the progression of MCI and AD through longitudinal assessments of MRI, PET, and other biomarkers, along with clinical and neuropsychological assessments. A detailed description of the ADNI cohort has been previously published^29^. Qualifying MCI subjects had: memory complaints, but no significant functional impairment; scored between 24 and 30 on the mini-mental status examination (MMSE); had a global clinical dementia rating (CDR) score of 0.5; a CDR memory score of 0.5 or greater; and objective memory impairment on the Wechsler Memory Scale – Logical Memory II test. Cognitively normal (CN) participants had: MMSE scores between 24 and 30; a global CDR of 0; and did not meet criteria for MCI or AD. Inclusion and diagnostic criteria, as well as procedures and protocols, for the ADNI studies can be found on http://www.adni-info.rg/Scientists/ADNIStudyProcedures.html.

For the current study, we included individuals with NC, MCI (both early and late MCI), and AD who had completed baseline sleep measures (Neuropsychiatric Inventory^30^/NPI Questionnaire [NPI-Q]), PET-Aβ data, and rs-fMRI scans.

### Standard Protocol Approvals, Registrations, and Patient Consents:

All procedures were approved by the Institutional Review Boards of all participating institutions. Written informed consent was obtained from every research participant according to the Declaration of Helsinki and the Belmont Report. For more up-to-date information, see www.adni-info.org.

### Neuropsychiatric Inventory (NPI)/Neuropsychiatric Inventory Questionnaire (NPIQ):

The presence of sleep disturbance was rated by an informant (e.g., caregiver or partner) and was assessed using the NPI and the NPI-Q. ADNI-1 used the NPI-Q while ADNI-GO/2 used the NPI. Both versions assess 12 neuropsychiatric symptoms, and the main difference between them is that NPI is conducted as a caregiver/informant interview whereas the NPI-Q is conducted in a questionnaire format. Severity and frequency ratings are highly correlated between NPI and NPI-Q^31^.

The 12 symptoms in the NPI/NPI-Q include hallucinations, delusions, agitation/aggression, dysphoric/depression, anxiety, irritability, disinhibition, euphoria, apathy, and aberrant motor behavior. The informant is first asked to rate the presence of each symptom within the past 1 month with “yes” or “no”, then if the answer is “yes,” is asked to rate severity (range 0–3). For the current study, sleep disturbance (SD) at baseline was determined to be present if the study partner endorsed having sleep disturbance (e.g., “Does the patient have difficulty sleeping? Is he/she up at night? Does he/she wander at night, get dressed, or disturb your sleep?”). It was coded as absent if not endorsed by the partner, and individuals without SD were categorized as Good Sleepers. Additionally, the total severity score was calculated for both NPI and NPI-Q by summing up the severity ratings for all domains except for sleep and nighttime behaviors. NPI or NPI-Q data were obtained from the Laboratory of Neuroimaging Image Data Archive (LONI IDA).

### Amyloid Positivity:

Florbetapir (AV45) PET images were processed by ADNI researchers (JAGUST LAB) as described in the previous publication^32^. Cortical summary regions were defined based on FreeSurfer v7.1.1. Florbetapir uptakes were calculated by dividing the cortical summary region by the whole cerebellum reference region. For cross-sectional florbetapir analyses, it is recommended to use a cutoff of 1.11 using the whole cerebellum reference region, which is equivalent to the upper 95% confidence interval above the mean of a group of young normal controls^33^ and transformed to the ADNI FreeSurfer (FS) pipeline initially using FS v5.3.^34^. The threshold was validated in the independent study^35^. The PET data used in this study were obtained from the ADNI files ‘UCBERKELEYAV45_04_26_22.csv’. A detailed description of PET acquisition, measurement, and quality control and preprocessing procedures were presented in http://adni.loni.usc.edu/methods/.

### rs-fMRI data analysis:

Functional data was preprocessed using fMRIPrep^30^; RRID:SCR_016216). Specifically, a reference volume and its skull-stripped version were generated using a custom methodology of fMRIPrep. Head-motion parameters with respect to the BOLD reference (transformation matrices, and six corresponding rotation and translation parameters) were estimated before any spatiotemporal filtering using mcflirt (FSL 5.0.9)^36^. BOLD runs were slice-time corrected using 3dTshift from AFNI 201 602 07^37^ (RRID:SCR_005927). The BOLD reference was then co-registered to the T1w reference using bbregister (FreeSurfer) which implements boundary-based registration^38^. Co-registration was configured with six degrees of freedom. The BOLD time-series were resampled into standard MNI152NLin2009cAsym space. Several confounding time-series were calculated based on the preprocessed BOLD: Frame-wise displacement (FWD) was calculated from the six motion parameters and root-mean-square difference (RMSD) of the BOLD percentage signal in the consecutive volumes. Contaminated volumes were then detected and classified as outliers by the criteria FWD > 0.5 mm or RMSD > 0.3% and replaced with new volumes generated by linear interpolation of adjacent volumes. The three global signals are extracted within the cerebrospinal fluid (CSF), the white matter masks. A bandpass filter with cut-off frequencies of 0.01 and 0.09 Hz was used. Finally, the covariates corresponding to head motion (6 realignment parameters), outliers, and the BOLD time series from the subject-specific white matter and CSF masks were used in the connectivity analysis as predictors of no interest, and were removed from the BOLD functional time series using linear regression.

ROI-to-ROI connectivity analysis was performed in CONN toolbox using 11 CONN resting state network nodes composing 3 networks (Default Mode Network (DMN): medial pre-frontal cortex (MPFC), precuneus cortex (PCC), bilateral lateral parietal (LP); Salience Network (SN): anterior cingulate cortex (ACC), bilateral anterior insula (AI), rostral pre-frontal cortex (RPFC), and supramarginal gyrus (SMG); Fronto-parietal Network (FP): bilateral lateral pre-frontal cortex (LPFC) and posterior parietal cortex (PPC)^39^. The mean BOLD time series was computed across all voxels within each ROI. Bivariate regression analyses were used to determine the linear association of the BOLD time series between each pair of regions for each subject. Both positive and negative correlations were examined. The resultant correlation coefficients were transformed into z-scores using Fisher’s transformation to satisfy normality assumptions. The within network-level FC was calculated as the average of the FCs within the networks of SN, DMN and FPN.

### Statistical Analysis:

Demographic characteristics between the control group and individuals with sleep disturbance were compared using the 2-sample independent T-tests (for continuous variables) and chi-square tests (for categorical variables). We first tested the association between sleep disturbance and rsFC using the linear regressions (“Main Effects”). Each linear regression includes each rsFC as the dependent variable and sleep disturbance as the independent variable after adjusting for age, sex, education, clinical status (CN, MCI, AD), sedatives hypnotics use, history of hypertension and total NPI as covariates. Then, Aβ was evaluated in the same model after replacing sleep disturbance with Aβ positivity. Finally, we evaluated the interactions between Aβ positivity and sleep disturbance by adding the Aβ × sleep disturbance interaction terms in the model (“Interaction Terms”). For the rsFC with significant Aβ positivity × sleep disturbance interaction (p < .05), we performed a post-hoc T-test to quantify the effect of sleep disturbance by Aβ positivity status. The multiple comparison corrections were performed within each hypothesis using False Discovery Rate (FDR) corrections. Regardless, the standardized beta coefficients and their 95% confidence intervals were reported in addition to p-values. All analyses were performed using R software (R Core Team, 2014, Vienna, Austria), and p-values < 0.05 were considered to indicate statistical significance.

### Data Availability:

ADNI datasets are available to the research community upon request at www.adni.loni.usc.edu. The processed imaging data are available for the qualified investigators upon request at seonjoo.lee@nyspi.columbia.edu.

## Results

The mean age of the entire study sample (N = 489) was 74.8 (SD 7.6) years, and 51 % were females. Sleep disturbance was reported in 87 individuals (17.8% of the study sample). Comparisons of the study sample by sleep disturbance groups indicated that there was a significantly greater proportion of females in the sleep disturbed group, compared to controls (Ps ≤ 0.04) (Table 1). Total NPI/NPIQ score was also greater in individuals with sleep disturbance, indicating greater neuropsychiatric symptoms (P < .001). Additionally, the sleep disturbance group had significantly less education and greater use of sleep medications (P ≤ 0.02). Other demographic characteristics, including age, clinical status, and Aβ positivity were not significantly different across groups, and both groups had comparable use of sedative/hypnotic medication use.

Presence of sleep disturbance was not significantly associated with any of the three intrinsic networks, after adjusting for covariates (Ps ≥ 0.18) (Table 2). Aβ positivity was not significantly associated with rsFC, but did have a negative association with the DMN at the trend-level significance (β=−0.03, P = 0.07). When the interaction term between sleep disturbance and Aβ was included, a significant interaction was found for the rsFC of the SN (β = 0.11, P = 0.006 [P = 0.018 after FDR corrections]) but not that of DMN or FPN, after adjusting for covariates (Table 2). Further examination revealed that sleep disturbance, in the presence of Aβ positivity, was associated with increased rsFC in the SN (Fig. 1), compared with the group without Aβ positivity.

Note: Separate models were employed to test the main effects of sleep disturbance and PET Aβ and the interaction term of the two variables (Model 1: main effect of sleep disturbance; Model 2: main effect of Aβ; Model 3: interactive effects of sleep disturbance and Aβ).

### Sensitivity Analyses

We conducted separate analyses without individuals with dementia, given the potential role of AD pathology on the association between sleep disturbance, Aβ positivity, and rsFC. In a sample with only CN and MCI (N = 407), our findings indicated that there was a significant interaction between sleep disturbance and Aβ positivity on the SN connectivity (β = 0.13, P = 0.002) but not on DMN or FPN, which are consistent with our main finding in all sample. This indicates that our main findings are not driven by unique characteristics of the AD group (i.e., neurodegenerative process) (Supplemental Table 1).

### Discussion

The present study examined the association between sleep disturbance, Aβ positivity, and resting-state functional connectivity within the SN, DMN and FPN. While we did not observe a significant direct association between sleep disturbance and rsFC within those three networks, there was a significant interaction between sleep disturbance and Aβ positivity on rsFC within the SN, suggesting that sleep disturbance was associated with aberrant salience network connectivity only when there was a presence of Aβ positivity.

Increased activation in SN is a well-established phenomenon related to AD pathology and sleep disorders. Hyperconnectivity in the SN is dependent on the stage of AD and has been particularly relevant to the preclinical stages of AD (e.g., presymptomatic or amnestic MCI)^9, 19, 40^. Interestingly, hyperactivity tends to wane during later stages of the disease and transition to a hypoconnectivity state^18, 19^. this paradoxically increased rsFC pattern has been noted in the DMN^17–19^ and also separately in the hippocampus^18^. SN hyperactivity is also noted with Aβ positivity^17^ and has been associated with Aβ-related increases in emotional sensitivity^41^. Clinically, elevated SN activation in AD manifest as neuropsychiatric symptoms, such as irritability, restlessness, anxiety, agitation, and suspiciousness, which are commonly seen in the progression of AD^9^.

The involvement of SN in sleep disorders has been commonly studied together with its role in emotion processing and regulation, such as detecting and perceiving emotionally salient information and initiating resources for appropriate behavioral response^42–44^. Sleep disturbance in the form of insomnia has been linked with increased activation in global functional connectivity, including the SN, FPN, DMN, and other cognitive control networks (dorsal attention network and visual network)^45, 46^. Hyperactivation in the SN may be conceptualized as a transdiagnostic marker of insomnia severity^47^, as it has been associated with common co-morbid psychiatric symptoms, such as depression^43, 48, 49^ and anxiety^50^. Within the SN, activity of the insula is particularly elevated in individuals with insomnia^51^. The insula’s role in SN is especially notable for detection of emotionally salient stimuli and could contribute to increased subjective alertness and negative affect in sleep disturbance. Additionally, the insula has been identified as a source of slow-wave activity measured by electroencephalogram (EEG) during sleep^52^, and the increased coactivation of the left insula with SN could disrupt generation of low-frequency EEG waves as part of sleep initiation^50^. The anterior cingulate cortex within the SN is associated with processing negative emotional stimuli^53^ and has been implicated in depressive symptoms associated with poor sleep^54^.

While sleep disturbance has been identified to cause significant damage to the brain (i.e., atrophy, neurotoxin build-up), hyperactivity in rsFC may represent “compensatory” connectivity in response to limit the clinical consequences of tissue damage in progress^55, 56^. Previous literature has indicated that hyperconnectivity in sleep disturbance is the brain’s hyperarousal state as a response to compensate for loss of energy^45^. In an insomnia model, it may also be true that somatic hyperarousal could lead to further cortical arousal and problems with sleep initiation and maintenance^57^.

Taken together, hyperconnectivity in the SN appears to represent insult to the brain that may be induced by both AD pathology and sleep disturbance. While sleep disturbance is being increasingly recognized as a modifiable risk factor for AD and its cognitive symptoms^58–60^, the current study clarified underlying neurobiological mechanisms and indicated that sleep disturbance may be associated with disrupted SN connectivity only when it interacts with established Aβ pathology. Based on this key finding, our data suggest that Aβ could induce AD-like rsFC profile in sleep disturbance. Functional connectivity of different brain networks (e.g., SN or DMN) has been shown to first increase with Aβ accumulation in the non-clinical stages while maintaining normal cognitive function, but eventually decreases as Aβ accumulates to abnormal (pathologic) levels^17^. This phenomenon is thought to reflect the earliest manifestation of cognitive reserve, the brain’s effort to compensate for emerging pathological processes^61^. Although our study did not assess other AD biomarkers or behavioral symptoms along with rsFC biomarkers, previous studies have found that Aβ augments AD processes (cognition and cortical thinning) that are associated with aberrant rsFC^62, 63^. Interestingly, our previous study also found that the presence of both sleep disturbance and Aβ burden was predictive of reduced cortical volume in AD regions and cognitive decline during a 5-year follow-up period, indicating that their interaction may help identifying individuals at an increased risk for AD (e.g., Aβ-positive individuals who also have sleep disorders)^64^.

The role of Aβ on the relationship between sleep disturbance and rsFC is an understudied area, and the current study used a large dataset from ADNI to demonstrate that Aβ plays a significant moderating role in brain activity that is induced by sleep disturbance. Based on our knowledge, this is the first study to examine this topic using a large dataset from ADNI that comprises individuals at various clinical stages. Nonetheless, there are also methodological limitations to this study that warrant careful interpretation. One major limitation is a lack of objective and standard sleep measure. Sleep disturbance was defined broadly (using the NPI/NPI-Q), regardless of its etiology (e.g., sleep apnea, insomnia, or other sleep disorders), and was based on informants’ observations. While only presence/absence of sleep disturbance was determined, we believe that such a dichotomous format may reflect the reality of screening conducted in the primary care setting and indicate that even quick and brief screening for sleep disturbance may provide insight into the risk of developing abnormal brain functioning. Previous studies from ADNI, NACC, and Harvard Aging Brain Study have also demonstrated clinical implications of poor/insufficient sleep in the community-based setting using a single-item subjective measure^65–67^. The cross-sectional design of this study is also a notable limitation that makes causal inferences difficult. Certainly, there is evidence supporting the impact of AD pathology on poor sleep^68^ and this makes the association between sleep disturbance and AD characteristics bi-directional; however, large-scale studies that utilized Mendelian randomization analyses demonstrated the impact of sleep on dementia risk^60, 69^, shedding more light on the hypothesis that sleep disturbance could cause AD-like brain changes and thus is a modifiable risk factor for AD. Future studies with longitudinal analyses may further elucidate the causal relationship between sleep disturbance and rsFC alterations.

## Conclusion

Findings from the current study imply that the presence of sleep disturbance is associated with rsFC alterations in the salience network when Aβ burden is present. Given that this aberrant brain connectivity may be signs of progression of Alzheimer’s pathology, sleep screening should be conducted routinely in older adults, particularly in those at risk of Aβ accumulation (e.g., individuals with mild cognitive impairment) to reduce sleep difficulties and slow the progression of AD.

## Figures and Tables

**Figure 2:**
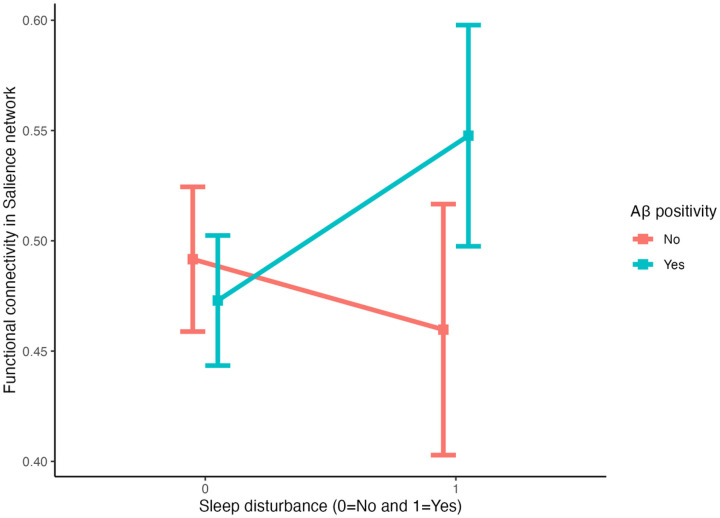
Interaction between sleep disturbance and amyloid beta positivity on the salience network connectivity Aβ=Amyloid-beta Note: Error bars represent the range of 95% Confidence Interval.

**Table 1:** Demographics of the study sample

	Control (N = 402)	Sleep Disturbance (N = 87)	Total (N = 489)	P Value
**Age** (Mean (SD))				0.66
Mean (SD)	74.73 (7.75)	75.05 (7.01)	74.78 (7.62)	
**Sex** (Women, n, %)	196 (48.8%)	53 (60.9%)	249 (50.9%)	0.04
**Education** (Mean (SD))	16.522 (2.550)	15.782 (2.522)	16.391 (2.558)	0.012
**Ethnicity** (n, %)				0.50
Hispanic	14 (3.5%)	5 (5.7%)	19 (3.9%)	
Not Hispanic	386 (96.0%)	82 (94.3%)	468 (95.7%)	
Unknown	2 (0.5%)	0 (0.0%)	2 (0.4%)	
**Race** (n, %)				0.42
White	358 (89.1%)	80 (92.0%)	438 (89.6%)	
Others	44 (10.9%)	7 (8.0%)	51 (10.4%)	
**Sleep Medication Use** (n, %)				0.02
No	363 (90.3%)	71 (81.6%)	434 (88.8%)	
Yes	39 (9.7%)	16 (18.4%)	55 (11.2%)	
**Hypertension** (n, %)	162 (40.3%)	43 (49.4%)	205 (41.9%)	0.12
**Cognitive Statics** (n, %)				0.19
Cognitively Normal	223 (55.5%)	39 (44.8%)	262 (53.6%)	
MCI	125 (31.1%)	34 (39.1%)	159 (32.5%)	
Dementia	54 (13.4%)	14 (16.1%)	68 (13.9%)	
**Total NPI/NPIQ score** (Mean (SD))	0.903 (1.801)	2.287 (3.053)	1.149 (2.143)	<0.001
**Aβ positivity** (n, %)				0.14
Below Cutoff	206 (51.2%)	37 (42.5%)	243 (49.7%)	
Above Cutoff	196 (48.8%)	50 (57.5%)	246 (50.3%)	

MCI = Mild Cognitive Impairment; NPI = Neuropsychiatric Inventory; NPIQ = Neuropsychiatric Inventory Questionnaire; Aβ = Amyloid-beta; APOE, SN = Salience Network; DMN = Default Mode Network; FPN = Frontoparietal Network.

**Table 2:** Main and Interaction Effects of Sleep Disturbance and Aβ

	SN			DMN			FPN		
Independent variables*	β	CI	P	β	CI	P	β	CI	P
Model 1:									
**Sleep Disturbance**	0.03	−0.01–0.07	0.18	0.01	−0.04–0.05	0.75	0.01	−0.04–0.06	0.66
Model 2:									
**PET Aβ**	0.0009	−0.03–0.03	0.96	−0.03	−0.07–0.0025	0.07	0.01	−0.03–0.05	0.79
Model 3:									
Sleep Disturbance	−0.03	−0.09–0.03	0.28	0.01	−0.06–0.07	0.86	−0.02	−0.10–0.05	0.56
PET Aβ	−0.02	−0.05–0.01	0.28	−0.03	−0.07 – 0.0045	0.08	−0.01	−0.05–0.04	0.81
**Sleep Disturbance × AP**	0.11	0.03–0.18	**0.006**	0.01	−0.08–0.09	0.90	0.06	−0.04–0.16	0.23

SN = Salience Network; DMN = Default Mode Network; FPN = frontoparietal network; PET = positron emission tomography; Aβ = Amyloid-beta

## References

[1] Rajan KB, Weuve J, Barnes LL, McAninch EA, Wilson RS, Evans DA. Population estimate of people with clinical Alzheimer’s disease and mild cognitive impairment in the United States (2020–2060). Alzheimers Dement 2021; 17(12): 1966–1975. 34043283 10.1002/alz.12362 PMC9013315

[2] He Y, Wang J, Wang L, Chen ZJ, Yan C, Yang H Uncovering intrinsic modular organization of spontaneous brain activity in humans. PLoS One 2009; 4(4): e5226. 19381298 10.1371/journal.pone.0005226 PMC2668183

[3] Raichle ME, MacLeod AM, Snyder AZ, Powers WJ, Gusnard DA, Shulman GL. A default mode of brain function. Proc Natl Acad Sci U S A 2001; 98(2): 676–682. 11209064 10.1073/pnas.98.2.676 PMC14647

[4] Chen BR, Kozberg MG, Bouchard MB, Shaik MA, Hillman EM. A critical role for the vascular endothelium in functional neurovascular coupling in the brain. J Am Heart Assoc 2014; 3(3): e000787. 24926076 10.1161/JAHA.114.000787 PMC4309064

[5] Buckner RL, Andrews-Hanna JR, Schacter DL. The brain’s default network: anatomy, function, and relevance to disease. Ann N YAcad Sci 2008; 1124: 1–38. 10.1196/annals.1440.011 18400922

[6] Lee JH, Delbruck T, Pfeiffer M. Training Deep Spiking Neural Networks Using Backpropagation. Front Neurosci 2016; 10: 508. 27877107 10.3389/fnins.2016.00508 PMC5099523

[7] Palmqvist S, Scholl M, Strandberg O, Mattsson N, Stomrud E, Zetterberg H Earliest accumulation of beta-amyloid occurs within the default-mode network and concurrently affects brain connectivity. Nat Commun 2017; 8(1): 1214. 29089479 10.1038/s41467-017-01150-x PMC5663717

[8] Li B, Liang F, Ding X, Yan Q, Zhao Y, Zhang X Interval and continuous exercise overcome memory deficits related to beta-Amyloid accumulation through modulating mitochondrial dynamics. Behav Brain Res 2019; 376: 112171. 31445975 10.1016/j.bbr.2019.112171

[9] Zhou J, Seeley WW. Network dysfunction in Alzheimer’s disease and frontotemporal dementia: implications for psychiatry. Biol Psychiatry 2014; 75(7): 565–573. 24629669 10.1016/j.biopsych.2014.01.020

[10] Zhao Q, Lu H, Metmer H, Li WXY, Lu J. Evaluating functional connectivity of executive control network and frontoparietal network in Alzheimer’s disease. Brain Res 2018; 1678: 262–272. 29079506 10.1016/j.brainres.2017.10.025

[11] Chand GB, Dhamala M. Interactions between the anterior cingulate-insula network and the fronto-parietal network during perceptual decision-making. Neuroimage 2017; 152: 381–389. 28284798 10.1016/j.neuroimage.2017.03.014

[12] Song J, Chang L, Zhou R. Test anxiety impairs filtering ability in visual working memory: Evidence from event-related potentials. J Affect Disord 2021; 292: 700–707. 34157666 10.1016/j.jad.2021.05.091

[13] Greicius MD, Flores BH, Menon V, Glover GH, Solvason HB, Kenna H Resting-state functional connectivity in major depression: abnormally increased contributions from subgenual cingulate cortex and thalamus. Biol Psychiatry 2007; 62(5): 429–437. 17210143 10.1016/j.biopsych.2006.09.020 PMC2001244

[14] Manoliu A, Riedl V, Zherdin A, Muhlau M, Schwerthoffer D, Scherr M Aberrant dependence of default mode/central executive network interactions on anterior insular salience network activity in schizophrenia. Schizophr Bull 2014; 40(2): 428–437. 23519021 10.1093/schbul/sbt037 PMC3932085

[15] Menon V, Uddin LQ. Saliency, switching, attention and control: a network model of insula function. Brain Struct Funct 2010; 214(5–6): 655–667. 20512370 10.1007/s00429-010-0262-0 PMC2899886

[16] Zhao Q, Sang X, Metmer H, Swati Z, Lu J, Alzheimer’s Disease NeuroImaging I. Functional segregation of executive control network and frontoparietal network in Alzheimer’s disease. Cortex 2019; 120: 36–48. 31228791 10.1016/j.cortex.2019.04.026

[17] Hahn A, Strandberg TO, Stomrud E, Nilsson M, van Westen D, Palmqvist S Association Between Earliest Amyloid Uptake and Functional Connectivity in Cognitively Unimpaired Elderly. Cereb Cortex 2019; 29(5):2173–2182. 30877785 10.1093/cercor/bhz020 PMC6458901

[18] Celone KA, Calhoun VD, Dickerson BC, Atri A, Chua EF, Miller SL Alterations in memory networks in mild cognitive impairment and Alzheimer’s disease: an independent component analysis. J Neurosci 2006; 26(40): 10222–10231. 17021177 10.1523/JNEUROSCI.2250-06.2006 PMC6674636

[19] Schultz AP Chhatwal JP Hedden T, Mormino EC, Hanseeuw BJ, Sepulcre J Phases of Hyperconnectivity and Hypoconnectivity in the Default Mode and Salience Networks Track with Amyloid and Tau in Clinically Normal Individuals. J Neurosci 2017; 37(16): 4323–4331. 28314821 10.1523/JNEUROSCI.3263-16.2017 PMC5413178

[20] Winer JR, Mander BA, Helfrich RF, Maass A, Harrison TM, Baker SL Sleep as a Potential Biomarker of Tau and beta-Amyloid Burden in the Human Brain. J Neurosci 2019; 39(32): 6315–6324. 31209175 10.1523/JNEUROSCI.0503-19.2019 PMC6687908

[21] Khazaie H, Veronese M, Noori K, Emamian F, Zarei M, Ashkan K Functional reorganization in obstructive sleep apnoea and insomnia: A systematic review of the resting-state fMRI. Neurosci Biobehav Rev 2017; 77: 219–231. 28344075 10.1016/j.neubiorev.2017.03.013 PMC6167921

[22] Nie X, Shao Y, Liu SY, Li HJ, Wan AL, Nie S Functional connectivity of paired default mode network subregions in primary insomnia. NeuropsychiatrDis Treat 2015; 11: 3085–3093. 10.2147/NDT.S95224 PMC4689284 26719693

[23] Dong D, Wang Y, Chang X, Luo C, Yao D. Dysfunction of Large-Scale Brain Networks in Schizophrenia: A Meta-analysis of Resting-State Functional Connectivity. Schizophr Bull 2018; 44(1): 168–181. 28338943 10.1093/schbul/sbx034 PMC5767956

[24] Ma J, Kim M, Kim J, Hong G, Namgung E, Park S Decreased functional connectivity within the salience network after two-week morning bright light exposure in individuals with sleep disturbances: a preliminary randomized controlled trial. Sleep Med 2020; 74: 66–72. 32841846 10.1016/j.sleep.2020.05.009

[25] Hedden T, Van Dijk KR, Becker JA, Mehta A, Sperling RA, Johnson KA Disruption of functional connectivity in clinically normal older adults harboring amyloid burden. J Neurosci 2009; 29(40): 12686–12694. 19812343 10.1523/JNEUROSCI.3189-09.2009 PMC2808119

[26] Mormino EC, Toueg TN, Azevedo C, Castillo JB, Guo W, Nadiadwala A Tau PET imaging with (18)F-PI-2620 in aging and neurodegenerative diseases. Eur J Nucl Med Mol Imaging 2021; 48(7): 2233–2244. 32572562 10.1007/s00259-020-04923-7 PMC7755737

[27] Kang JE, Lim MM, Bateman RJ, Lee JJ, Smyth LP, Cirrito JR Amyloid-beta dynamics are regulated by orexin and the sleep-wake cycle. Science 2009; 326(5955): 1005–1007. 19779148 10.1126/science.1180962 PMC2789838

[28] Wilckens KA, Tudorascu DL, Snitz BE, Price JC, Aizenstein HJ, Lopez OL Sleep moderates the relationship between amyloid beta and memory recall. Neurobiol Aging 2018; 71: 142–148. 30138767 10.1016/j.neurobiolaging.2018.07.011 PMC6416050

[29] Petersen RC, Aisen PS, Beckett LA, Donohue MC, Gamst AC, Harvey DJ Alzheimer’s Disease Neuroimaging Initiative (ADNI): clinical characterization. Neurology 2010; 74(3): 201–209. 20042704 10.1212/WNL.0b013e3181cb3e25 PMC2809036

[30] Esteban O, Markiewicz CJ, Blair RW, Moodie CA, Isik AI, Erramuzpe A fMRIPrep: a robust preprocessing pipeline for functional MRI. Nat Methods 2019; 16(1): 111–116. 30532080 10.1038/s41592-018-0235-4 PMC6319393

[31] Cummings JL, Mega M, Gray K, Rosenberg-Thompson S, Carusi DA, Gornbein J. The Neuropsychiatric Inventory: comprehensive assessment of psychopathology in dementia. Neurology 1994; 44(12): 2308–2314. 7991117 10.1212/wnl.44.12.2308

[32] Jagust WJ, Landau SM, Koeppe RA, Reiman EM, Chen K, Mathis CA The Alzheimer’s Disease Neuroimaging Initiative 2 PET Core: 2015. Alzheimers Dement2015; 11(7): 757–771. 10.1016/j.jalz.2015.05.001 PMC4510459 26194311

[33] Landau SM, Breault C, Joshi AD, Pontecorvo M, Mathis CA, Jagust WJ Amyloid-beta imaging with Pittsburgh compound B and florbetapir: comparing radiotracers and quantification methods. J Nucl Med 2013; 54(1): 70–77. 23166389 10.2967/jnumed.112.109009 PMC3747730

[34] Joshi AD, Pontecorvo MJ, Clark CM, Carpenter AP, Jennings DL, Sadowsky CH Performance characteristics of amyloid PET with florbetapir F 18 in patients with alzheimer’s disease and cognitively normal subjects. J Nucl Med 2012; 53(3): 378–384. 22331215 10.2967/jnumed.111.090340

[35] Royse SK, Minhas DS, Lopresti BJ, Murphy A, Ward T, Koeppe RA Validation of amyloid PET positivity thresholds in centiloids: a multisite PET study approach. Alzheimers Res Ther2021; 13(1): 99. 10.1186/s13195-021-00836-1 PMC8111744 33971965

[36] Jenkinson M, Bannister P, Brady M, Smith S. Improved optimization for the robust and accurate linear registration and motion correction of brain images. Neuroimage 2002; 17(2): 825–841. 12377157 10.1016/s1053-8119(02)91132-8

[37] Cox RW, Hyde JS. Software tools for analysis and visualization of fMRI data. NMR Biomed 1997; 10(4–5): 171–178. 9430344 10.1002/(sici)1099-1492(199706/08)10:4/5<171::aid-nbm453>3.0.co;2-l

[38] Greve DN, Fischl B. Accurate and robust brain image alignment using boundary-based registration. Neuroimage2009; 48(1): 63–72. 10.1016/j.neuroimage.2009.06.060 PMC2733527 19573611

[39] Whitfield-Gabrieli S, Nieto-Castanon A. Conn: a functional connectivity toolbox for correlated and anticorrelated brain networks. Brain Connect 2012; 2(3): 125–141. 22642651 10.1089/brain.2012.0073

[40] Badhwar A, Tam A, Dansereau C, Orban P, Hoffstaedter F, Bellec P Resting-state network dysfunction in Alzheimer’s disease: A systematic review and meta-analysis. Alzheimers Dement (Amst) 2017; 8: 73–85. 28560308 10.1016/j.dadm.2017.03.007 PMC5436069

[41] Fredericks CA, Sturm VE, Brown JA, Hua AY, Bilgel M, Wong DF Early affective changes and increased connectivity in preclinical Alzheimer’s disease. Alzheimers Dement (Amst) 2018; 10: 471–479. 30302368 10.1016/j.dadm.2018.06.002 PMC6174255

[42] Menon V. Large-scale brain networks and psychopathology: a unifying triple network model. Trends Cogn Sci2011; 15(10): 483–506. 10.1016/j.tics.2011.08.003 21908230

[43] Hamilton JP, Glover GH, Bagarinao E, Chang C, Mackey S, Sacchet MD Effects of salience-network-node neurofeedback training on affective biases in major depressive disorder. Psychiatry Res Neuroimaging 2016; 249: 91–96. 26862057 10.1016/j.pscychresns.2016.01.016 PMC4803612

[44] Seeley WW. Mapping Neurodegenerative Disease Onset and Progression. Cold Spring Harb Perspect Biol 2017; 9(8). 10.1101/cshperspect.a023622 PMC5538416 28289062

[45] Yu S, Guo B, Shen Z, Wang Z, Kui Y, Hu Y The imbalanced anterior and posterior default mode network in the primary insomnia. J Psychiatr Res 2018; 103: 97–103. 29804003 10.1016/j.jpsychires.2018.05.013

[46] Cheng JC, Anzolin A, Berry M, Honari H, Paschali M, Lazaridou A Dynamic Functional Brain Connectivity Underlying Temporal Summation of Pain in Fibromyalgia. Arthritis Rheumatol 2022; 74(4): 700–710. 34725971 10.1002/art.42013

[47] Van Someren EJW. Brain mechanisms of insomnia: new perspectives on causes and consequences. Physiol Rev 2021; 101 (3): 995–1046. 32790576 10.1152/physrev.00046.2019

[48] Hilland E, Landro NI, Harmer CJ, Maglanoc LA, Jonassen R. Within-Network Connectivity in the Salience Network After Attention Bias Modification Training in Residual Depression: Report From a Preregistered Clinical Trial. Front Hum Neurosci 2018; 12: 508. 30622463 10.3389/fnhum.2018.00508 PMC6308203

[49] Avery JA, Drevets WC, Moseman SE, Bodurka J, Barcalow JC, Simmons WK. Major depressive disorder is associated with abnormal interoceptive activity and functional connectivity in the insula. Biol Psychiatry 2014; 76(3): 258–266. 24387823 10.1016/j.biopsych.2013.11.027 PMC4048794

[50] Van Someren EJ. Doing with less sleep remains a dream. Proc Natl Acad Sci U S A 2010; 107(37): 16003–16004. 20810921 10.1073/pnas.1011249107 PMC2941300

[51] Chen HC, Su TP Chou P A nine-year follow-up study of sleep patterns and mortality in community-dwelling older adults in Taiwan. Sleep 2013; 36(8): 1187–1198. 23904679 10.5665/sleep.2884 PMC3700716

[52] Murphy M, Riedner BA, Huber R, Massimini M, Ferrarelli F, Tononi G. Source modeling sleep slow waves. Proc Natl Acad Sci U S A 2009; 106(5): 1608–1613. 19164756 10.1073/pnas.0807933106 PMC2635823

[53] Grabenhorst F, Rolls ET. Value, pleasure and choice in the ventral prefrontal cortex. Trends Cogn Sci 2011; 15(2): 56–67. 21216655 10.1016/j.tics.2010.12.004

[54] Cheng H, Gurland BJ, Maurer MS. Self-reported lack of energy (anergia) among elders in a multiethnic community. J Gerontol A Biol Sci Med Sci 2008; 63(7): 707–714. 18693225 10.1093/gerona/63.7.707

[55] Agosta E, Lazzeri S, Orlandi P, Figus M, Fioravanti A, Di Desidero T Pharmacogenetics of antiangiogenic and antineovascular therapies of age-related macular degeneration. Pharmacogenomics2012; 13(9): 1037–1053. 10.2217/pgs.12.77 22838951

[56] Dickerson BC, Salat DH, Bates JF, Atiya M, Killiany RJ, Greve DN Medial temporal lobe function and structure in mild cognitive impairment. Ann Neurol2004; 56(1): 27–35. 10.1002/ana.20163 PMC4335689 15236399

[57] Killgore WD, Schwab ZJ, Kipman M, Deldonno SR, Weber M. Insomnia-related complaints correlate with functional connectivity between sensory-motor regions. Neuroreport 2013; 24(5): 233–240. 23399993 10.1097/WNR.0b013e32835edbdd

[58] Bubu OM, Brannick M, Mortimer J, Umasabor-Bubu O, Sebastiao YV, Wen Y Sleep, Cognitive impairment, and Alzheimer’s disease: A Systematic Review and Meta-Analysis. Sleep 2017; 40(1). 10.1093/sleep/zsw032 28364458

[59] Pase MP, Himali JJ, Grima NA, Beiser AS, Satizabal CL, Aparicio HJ Sleep architecture and the risk of incident dementia in the community. Neurology 2017; 89(12): 1244–1250. 28835407 10.1212/WNL.0000000000004373 PMC5606917

[60] Henry A, Katsoulis M, Masi S, Fatemifar G, Denaxas S, Acosta D The relationship between sleep duration, cognition and dementia: a Mendelian randomization study. Int J Epidemiol 2019; 48(3): 849–860. 31062029 10.1093/ije/dyz071 PMC6659373

[61] Stern Y. Cognitive reserve in ageing and Alzheimer’s disease. Lancet Neurol 2012; 11(11): 1006–1012. 23079557 10.1016/S1474-4422(12)70191-6 PMC3507991

[62] Buckley RF, Hanseeuw B, Schultz AP Vannini P Aghjayan SL, Properzi MJ Region-Specific Association of Subjective Cognitive Decline With Tauopathy Independent of Global beta-Amyloid Burden. JAMA Neurol2017; 74(12): 1455–1463. 10.1001/jamaneurol.2017.2216 PMC5774633 28973551

[63] Hampton OL, Buckley RF, Manning LK, Scott MR, Properzi MJ, Pena-Gomez C Resting-state functional connectivity and amyloid burden influence longitudinal cortical thinning in the default mode network in preclinical Alzheimer’s disease. Neuroimage Clin 2020; 28: 102407. 32942175 10.1016/j.nicl.2020.102407 PMC7498941

[64] Kim H, Levine A, Cohen D, Gehrman P, Zhu X, Devanand DP The Role of Amyloid, Tau, and APOE Genotype on the Relationship Between Informant-Reported Sleep Disturbance and Alzheimer’s Disease Risks. J Alzheimers Dis 2022; 87 : 1567–1580. 35491776 10.3233/JAD-215417 PMC9644449

[65] Mecca AP, Michalak HR, McDonald JW, Kemp EC, Pugh EA, Becker ML Sleep Disturbance and the Risk of Cognitive Decline or Clinical Conversion in the ADNI Cohort. Dement Geriatr Cogn Disord 2018; 45(3–4): 232–242. 29886490 10.1159/000488671 PMC6178799

[66] Goukasian N, Hwang KS, Romero T, Grotts J, Do TM, Groh JR Association of brain amyloidosis with the incidence and frequency of neuropsychiatric symptoms in ADNI: a multisite observational cohort study. BMJ Open 2019; 9(12): e031947. 10.1136/bmjopen-2019-031947 PMC6937083 31857304

[67] Winer JR, Deters KD, Kennedy G, Jin M, Goldstein-Piekarski A, Poston KL Association of Short and Long Sleep Duration With Amyloid-beta Burden and Cognition in Aging. JAMA Neurol 2021; 78(10): 1187–1196. 34459862 10.1001/jamaneurol.2021.2876 PMC8406215

[68] Wang C, Holtzman DM. Bidirectional relationship between sleep and Alzheimer’s disease: role of amyloid, tau, and other factors. Neuropsychopharmacology 2020; 45(1): 104–120. 31408876 10.1038/s41386-019-0478-5 PMC6879647

[69] Lu K, Nicholas JM, James SN, Lane CA, Parker TD, Keshavan A Increased variability in reaction time is associated with amyloid beta pathology at age 70. Alzheimers Dement (Amst) 2020; 12(1): e12076. 32789161 10.1002/dad2.12076 PMC7416668

